# Green metal nanotechnology in monogastric animal health: current trends and future prospects — A review

**DOI:** 10.5713/ab.24.0506

**Published:** 2024-10-28

**Authors:** Sungyeon Chin, Mohammad Moniruzzaman, Elena Smirnova, Do Thi Cat Thoung, Anjana Sureshbabu, Adhimoolam Karthikeyan, Dong I. Lee, Taesun Min

**Affiliations:** 1 Department of Animal Biotechnology, Jeju International Animal Research Center (JIA) & Sustainable Agriculture Research Institute (SARI), Jeju National University, Jeju 63243, Korea; 2 Subtropical Horticulture Research Institute, Jeju National University, Jeju 63243, Korea; 3 Department of Biomedical Engineering, Johns Hopkins University School of Medicine, Baltimore, MD 21205, USA; 4 Department of Animal Biotechnology, Bio-resources Computing Research Center, Sustainable Agriculture Research Institute (SARI), Jeju National University, Jeju 63243, Korea

**Keywords:** Green Synthesis, Health, Immunity, Metals, Monogastric Animal, Nanotechnology

## Abstract

Green nanotechnology is an emerging field of research in recent decades with rapidly growing interest. This integrates green chemistry with green engineering to avoid using toxic chemicals in the synthesis of organic nanomaterials. Green nanotechnology would create a huge potential for the use of nanoparticles for more sustainable utilization in improving animal health. Nanoparticles can be synthesised by physical, chemical and biological processes. Traditional methods for physical and chemical synthesis of nanoparticles are toxic to humans, animals and environmental health, which limits their usefulness. Green synthesis of nanoparticles via biological processes and their application in animal health could maximize the benefits of nanotechnology in terms of enhancing food animal health and production as well as minimize the undesirable impacts on Planetary Health. Recent advances in nanotechnology have meant different nanomaterials, especially those from metal sources, are now available for use in nanomedicine. Metal nanoparticles are one of the most widely researched in green nanotechnology, and the number of articles on this subject in food animal production is growing. Therefore, research on metal nanoparticles using green technologies have utmost importance. In this review, we report the recent advancement of green synthesized metal nanoparticles in terms of their utilization in monogastric animal health, elucidate the research gap in this field and provide recommendations for future prospects.

## INTRODUCTION

Nanotechnology (NT) refers to the technology of understanding and controlling materials in the range of 1 to 100 nanometers, hereafter nanoparticles (NPs) [1]. It applies research in creating or modifying new objects with altering the particle’s physical and chemical properties [2,3]. The characteristics of NT enable innovative applications across various fields. NPs have a large surface area to volume ratio, and their small size makes them highly biocompatible and favorable for transport to specific organs [4–6]. They also play a role in stabilizing nutrients and enhancing cell absorption, mitigating risks associated with directly adding bioactive substances to feed. In recent years, NT has gained attention in a wide range of fields, including medicine, food, chemistry, agriculture, etc. [7–9]. The NT provides transformative solutions for biological systems, holding significant potential for enhancing animal farming systems [8]. Furthermore, NPs enable targeted drug delivery to specific sites in organisms and enhance the effectivity of the drugs [10,11]. Feed supplementation using NT can significantly increase the body's absorption of nutrients and enable precise delivery of specific nutrients, unlike conventional feeds that are poorly absorbed and easily degraded [12,13]. Many studies have reported the effectiveness of adding NPs to animal feed to improve weight gain and nutrition [14–17]. Feed and nutrient delivery systems utilizing NT were reported to play an important role in enhancing the productivity and efficiency of livestock farming, improving animal health and food quality [18]. For this instance, green nanotechnology (GNT) is an important factor that can further develop these innovations. The GNT offers safer, less toxic, and more energy-efficient synthetic methods compared to conventional chemical synthesis [19]. Feed and nutrient delivery systems utilizing GNT may contribute to increase the sustainability and efficiency of livestock farming, and to fulfill our responsibility to animals and the environment [20]. With continued research and evaluation, this technology could change the future prospects of agriculture and provide a safe and effective solution.

## EMERGENCE OF GREEN METAL NANOTECHNOLOGY

The main methods for synthesizing nanomaterials are physical and chemical methods [21]. The electrochemical method, sol-gel, precipitation, hydrothermal approach, chemical bath deposition, and chemical reduction methods can be referred to as chemical methods [22]. Nanoparticles are commonly produced by reducing metal ions to neutral NPs using potentially harmful reducing agents [23]. The costly and toxic chemicals used as stabilizing and reducing agents during the synthesis of these NPs may restrict their applications [24,25]. The consequences are linked to the toxicity from the use of hazardous chemicals in synthesis, as well as concerns over environmental pollution due to the generation of significant amounts of toxic by-products and the high cost associated with elevated reaction conditions [25,26]. Traditional chemical and physical synthesis methods are expensive, harmful to the environment, and unsafe [26]. Therefore, utilizing plant extracts for metal NPs synthesis offers an environmentally friendly, safe, and economical alternative [27–41].

The term ‘green metal NT’ refers to the synthesis, characterization, and biological effects evaluation of NPs derived from plants such as silver, copper, gold, iron, zinc, and selenium [37,40]. Plant extracts play a major role as reducing and capping agents for metal NP synthesis [27,29,32,35]. Notable advantage of green metal NT is the utilization of plant-derived phytochemical constituents such as polyphenols, alkaloids, terpenoids [38]. The green metal NT is essential to avoid the production of unwanted or harmful by-products, ensuring the establishment of reliable, sustainable, and environmentally friendly synthesis of NPs [40]. Green synthesis provides an eco-friendly, non-toxic, and efficient method for synthesizing metal NPs. These NPs do not use harmful chemicals, exhibit excellent stability, and prevent aggregation, offering advantages for various applications across different fields [41].

Currently, metal NPs have been synthesized through various physical and chemical methods, as evidenced by several studies [28]. However, most of these methods are capital-intensive, toxic, environmentally unsafe, and have low productivity. Therefore, there is a current necessity in NT to adopt alternative green routes for metal NP synthesis (Table 1).

## PLANT BASED METAL NANOPARTICLE SYNTHESIS

Plant-based metal NP synthesis offers an eco-friendly, cost-effective, and safe approach to producing NPs with unique properties. Plant-based biological molecule extracts serve as the foundation for NP production, surpassing conventional chemical technique. Plants harbor various unique compounds that aid in the synthesis process and accelerate the synthesis rate [36,37]. The size and distribution of NPs derived from plant extracts are significantly influenced by the biological constituents present in the extracts. The potent reducing biomolecules within the extract expedite reactions, allowing for the formation of smaller NPs [41]. Utilizing plants as a medium for NPs production offers advantages over alternative biological methods. The employments of antioxidant agents like plant extracts, natural chemicals, vitamins, and minerals in NPs synthesis has resulted in favorable outcomes. Different types of plant materials can be utilized to generate metal NPs due to their accessibility, affordability, environmental friendliness, and capacity to produce NPs of diverse sized and shapes through biosynthesis. This is feasible because plant components contribute distinct properties to the NP formation process.

Unlike physical or chemical methods, which require extensive energy consumption or involve the use of toxic solvent, plant based synthesis achieves rapid reduction of metal ions at room temperature [31]. Fungi, among biological elements, exhibit significant resilience and possess the capability to accumulate metals, making them essential factors in NP synthesis [19]. Nanoparticle production is possible with each living organism's specific biochemical processing abilities. Nanoparticles can only be synthesized by certain biological organisms because of their enzyme activity and metabolic processes. Therefore, to produce NPs with well-defined features such as size and form, it is necessary to carefully select the appropriate biological entity [36].

## SYNTHESIS AND APPLICATION OF GREEN METAL NANOTECHNOLOGY IN ANIMAL NUTRITION

### Gold nanoparticles

Gold nanoparticles (AuNPs) hold tremendous potential to revolutionize drug delivery by enabling more targeted, efficient, and safe therapies. Furthermore, they are actively researched as safe and reliable biomaterial substitutes for chemical agents in various applications [53]. Ongoing research continues to explore the full potential of AuNPs in various biomedical applications, including cancer treatment, gene therapy, and vaccine delivery [54,55]. Plant extracts have been utilized in various studies for the green synthesis of AuNPs [56–61]. Rajakumar et al [56] reported the green synthesis of AuNPs through the reduction of chloroauric acid (HAuCl_4_) using *Eclipta prostrata* leaf extract, and the synthesized AuNPs were found to affect antioxidant and cytotoxic activities. Boruah et al [57] reported that green AuNPs using *Moringa oleifera* leaf extract was less cytotoxic and helped in regeneration of neuronal cells in animal.

### Application of green synthesized gold nanoparticles in monogastric animal health

The utilization of plant cells for the eco-friendly synthesis of AuNPs represents a significant and environmentally sustainable approach. This method involves the reduction of metal ions to elemental metals and their stabilization, with plant cell-method synthesis playing a central role across diverse applications. Ma et al [58] investigated the preventive effects of green decorated AuNPs on magnetic NPs mediated by *Calendula* flower extract in streptozotocin-induced gestational diabetes mellitus rats. Treatment with Fe_3_O_4_/AuNPs reduced the elevated levels of aspartate transferase and alanine phosphatase enzymes in rats and showed positive results in reducing fasting blood glucose in diabetic rats. This demonstrates the hepatoprotective and antidiabetic properties of AuNPs, suggesting their potential use as hepatoprotective and antidiabetic supplements for preventing gestational diabetes mellitus. Akkam et al [59] demonstrated that the acute and chronic adverse effects of AuNPs synthesized using *Ziziphus zizyphus* leaf extract in mice. The results revealed that the AuNPs did not increase in concentration in the blood and heart, but there were proportional increases in gold content observed in the liver and kidney. These findings indicate that AuNPs synthesized at a dose of 1 mg/kg may induce adverse effects in the kidneys. Bommavaram et al [60] investigated the antioxidant potential of AuNPs synthesized through *Bacopa monniera* (BmGNPS). The antioxidant activity of BmGNPS was evaluated against aluminum-induced oxidative stress in mice. The results showed that exposure to aluminum acetate led to an increase in thiobarbituric acid reactive substances levels and a decrease in superoxide dismutase (SOD), catalase, and glutathione peroxidase activities, indicating oxidative stress. It was concluded that BmGNPs possess the ability to inhibit aluminum-induced oxidative stress in mice. Zugravu et al [61] found that administration improved aortic function, mitigated high-fat diet-induced aortic wall changes and liver injury, and enhanced antioxidant capacity in rats (Table 2).

### Silver nanoparticles

Silver is a transition metal with high electrical conductivity, thermal conductivity, and reflectivity. Its high surface area, tunable optical properties, and good antimicrobial activity make it suitable for a wide range of applications. Green synthesized silver nanoparticles (AgNPs) have low synthesis costs and have the bioreduction potential of plant-derived NPs [62]. Green synthesized AgNPs play an important role in biological functions, especially as an alternative to antibiotics in animal production [63].

### Application of green synthesized silver nanoparticles in monogastric animal health

El-Abd et al [64] study examined the effects of green AgNPs fabricated from *Corallina elongata* extract and/or coated with acetic acid on the performance and immune response of Ross broilers. The results showed that the groups treated with AgNPs exhibited increased body weight and antimicrobial activity. These findings suggest that green synthesized AgNPs may enhance productivity when administered to poultry and show promise as an alternative to antibiotics. Lohakare and Abdel-Wareth [65] demonstrated that the results of supplementing poultry with oregano bioactive lipid compounds (OBLC) and AgNPs showed improved growth and survival rates in the group treated with AgNPs. Additionally, lower mortality rates and positive outcomes in liver function and dressing percentage were observed in the groups treated with OBLC and AgNPs. Contrary to this, there was also a toxicity evaluation study on AgNPs. According to Farag et al [66] toxicity assessment of traditionally synthesized AgNPs in Nile tilapia revealed that AgNPs exerted harmful effects on physiological health and antioxidant defense systems in the group administered with 1.98 mg/L. Furthermore, Al-Sultan et al [67] reported that broilers with chemically synthesized AgNPs showed cytotoxicity in a dose dependent manner. These results show that green synthesized AgNPs may be nutritionally safer and more efficient than chemical synthesis methods. Therefore, additional research on the toxicity of AgNPs appears to be necessary. Green synthesized AgNPs can be used as supplements in aquaculture. Mansour et al [68] exposed Nile tilapia to various doses of green-synthesized AgNPs for toxicity assessment. Results showed that exposure to doses exceeding 3.31 mg/L suppressed the expression of antioxidant genes and reduced immune enzyme activity. This indicates that AgNPs at concentrations of 3.31 mg/L or higher can induce toxicity in aquaculture (Table 3).

### Zinc nanoparticles

Zinc, an essential trace metal, plays vital roles in enzyme activity within metabolic pathways, hormone regulation, and immune system function [69]. Zinc plays crucial role in cell division, aiding in the resilience of respiratory and digestive mucous membranes and fostering cell proliferation. Furthermore, zinc contributes significantly to the body’s antioxidant enzyme system, which helps counteract oxidative stress. However, insufficient zinc levels can compromise liver function and immune responses, leading to various disease [70]. Due to the essential nature and various advantages of zinc, research on green synthesized nano zinc has been actively conducted recently [65–70].

### Application of green synthesized zinc nanoparticles in monogastric animal health

Hidayat et al [71] reported that synthesizing nano zinc through guava leaf extract and feeding it to broiler chickens resulted in the highest body weight and SOD increase at 90 mg/kg. It also showed lower bacterial counts. Through this, it was concluded that adding nano zinc up to 90 mg Zn/kg to broiler chicken feed provides maximum benefits. In addition, Lail et al [72] demonstrated that the supplementation of ZnO-NPs synthesized from *Nigella sativa* improved the growth performance in *Eimeria tenella*-infected broilers. The green synthesized ZnO-NPs-feed group exhibited anti-inflammatory and antioxidant activities, suggesting the potential use as an alternative treatment for diseases. Kim et al [73] reported that zinc oxide nanoparticles (ZnONPs) have toxic effects when used in high doses. Albeit, it was stated that ZnONPs supplementation had a significant effect on improving bone quality and physiological function in broilers [74,75]. Similar studies revealed that green synthesized ZnONPs could not only overcome zinc deficiency in fish, but also reduce liver damage more safely than chemically synthesized methods [76,77]. Dukare at el [75] explained that a comparative study between inorganic nano-zinc and green nano-zinc revealed that 80 ppm of green nano-zinc improved the antioxidant status of zinc, reduced meat cholesterol, fat content, and lipid oxidation, and increased bone dimensions and mineralization. Thangapandiyan and Monika [76] investigated the growth effects of ZnONP-supplemented diets in fingerlings of freshwater fish, *Labeo rohita*. The results revealed that the group fed with a supplementation of 10 mg/kg exhibited significantly improved growth performance and increased activities of blood and digestive enzymes. Furthermore, Ghafarifarsani et al [77] reported that green synthesized zinc nanoparticles (ZnNPs) positively impacted the changes in biochemical parameters when fed to gold fish. These suggest that green synthesized ZnO-NPs can lead to a significant enhancements in the growth and metabolic functions of fish (Table 4).

### Selenium nanoparticles

Selenium is an important dietary trace element for life and is essential for the growth and function of surviving cells [78]. Selenium is naturally found in both inorganic and organic states known as selenate, selenite, elemental selenium and selenide. With inorganic forms known as selenates and selenites [78,79]. Selenium compounds commonly exist in four oxidation states in nature form [5]. Selenium, a vital mineral for both humans and animals, acts as an antioxidant when consumed in small amount. Notably Se-NPs derived from plant sources exhibit remarkable biocompatibility, bioavailability, and low toxicity, further promoting their eco-friendliness and suitability for diverse applications [74]. Additionally, they offer various health benefits, including enhanced growth performance and increased resistance to diseases [75].

### Application of green synthesized selenium nanoparticles in monogastric animal health

Bami et al [80] compared the effects of green-synthesized nano-selenium (GNS) and selenium selenite on broiler chickens, and the results showed better outcomes with green-synthesized nano-selenium. While there was no significant impact on growth, GNS resulted in higher meat selenium content, improved intestinal microflora, intestinal morphology, and immune response compared to selenium chloride. It was concluded that GNS had a greater influence on broiler chickens. Dukare et al [81] found that feeding broiler chickens with 0.25 ppm of organic nano-selenium resulted in improved growth, immune response, lymphoid organ development, nitrogen digestibility coefficient, and serum antioxidant activity. However, the research revealed little difference between green-synthesized NPs and chemically synthesized NPs. The researchers opined that the eco-friendly green nano Se can be used in place of chemically synthesized nano Se. Ibrahim et al [82] reported that supplemented various concentrations of dietary green synthesized nano-selenium from scent leaf, *Ocimum gratissimum* extract, revealed significant improvements in growth and immunological parameters. This indicates that the growth performance and immunity of broiler chickens can be enhanced by consuming diets supplemented with green synthesized nano selenium. In addition, Naz et al [83] reported that the lower dose of 0.2 mg/kg may have beneficial effects on growth. however, the higherdose of 0.4 mg/kg not only negatively impacts growth but also leads to histopathological alterations in major organs of the body and DNA damage as well. Collectively, green synthesized nano-selenium possesses various advantages such as low toxicity and biocompatibility. If administered in appropriate doses according to the animal, it could potentially be used as a future animal additive (Table 5).

### Copper nanoparticles

Copper is an essential element for both humans and animals, playing important roles in various physiological and biochemical processes [84]. Copper nanoparticles (CuNPs), with sizes smaller than 100 nanometers, possess numerous advantages such as electrical conductivity, thermal conductivity, antimicrobial properties, catalytic activity, and surface modification capability. Moreover, numerous studies have reported the beneficial effects of copper on promoting animal growth, exhibiting antimicrobial properties, and modulating immune responses [85].

### Application of green synthesized copper nanoparticles in monogastric animal health

Mohamed et al [86] investigated the effects of green synthesized nano-copper oxide (nano-CuO) on growth performance and serum biochemistry of broiler chickens. The results from the broiler experiment revealed that the group treated with nano-CuO at 8 mg/kg showed improved body weight gain and feed conversion ratio, along with enhanced liver and kidney functions as evidenced by serum metabolites. Khatami et al [87] demonstrated that CuNPs synthesized from aqueous extract of *C. spinosa* fruit showed no toxicity in the liver of the studied mice, and no significant toxicity was observed in their hematological paramerters. Dukare et al [88] experimented with various levels and sources of copper in broiler chickens, and the group fed with 16 ppm of green nano copper (GNC) showed superior effects and immune response. Additionally, it was noted that green synthesized CuNPs enhanced cellular immunity compared to other forms of copper. In addition, Kumar et al [89] found that dietary green synthesized nano copper enhanced immunity in terms of blood antibody profile, antioxidant status and immune response in Vanaraja chickens. Naz et al [90] reported that chemically synthesized CuNPs exhibited dose-dependent toxic effects on the liver in rats compared to green-synthesized CuNPs. Therefore, it was suggested that green-synthesized CuNPs show potential for pharmacological application due to their biocompatible nature (Table 6).

### Manganese nanoparticles

Manganese plays a crucial role in various physiological process, including metabolism, skeletal development, enzyme function, and immune system responses [91,92]. Manganese is essential for a range of physiological functions, such as bone mineralization, energy metabolism, protein metabolism, glycosaminoglycan synthesis, protection against free radicals, and metabolic regulation [91,92]. Additionally, it serves as a component in numerous metalloenzymes [91]. While dietary manganese deficiency typically has minimal impact on immunity, it can hinder the body’s humoral immune response. Manganese exhibits unique physical and chemical properties such as high surface area and irregular crystal morphology [91]. Manganese is essential, but maintaining appropriate levels of intracellular manganese is important as excess manganese can be toxic [92].

### Application of green synthesized manganese nanoparticles in monogastric animal health

Helbawi et al [93] postulated that using green tomato extract, manganese dioxide nanoparticles (MnO_2_NPs) were added as a supplement to broiler chicken feed, and nutritional performance experiments revealed that MnO_2_ NPs did not have a negative impact on broiler growth and antioxidant status. In groups fed with doses of 66 mg/kg and 72 mg/kg, increased SOD activity was observed in both serum and liver tissues, along with positive effects on growth performance. Asaikkutti et al [94] reported that using *Ananas comosus* (L.) peel extract, the researchers’ synthesized stable Mn_3_O_4_ NPs and tested their efficacy by feeding them to freshwater prawns, *Macrobrachium rosenbergii*. Through the study, it was revealed that manganese nanoparticles (MnNPs) enhanced weight gain and growth performance. It was stated that supplementation with 16 mg/kg of MnO_2_NPs could lead to improved survival, growth, and production. Verma et al [95] demonstrated that the green synthesis of MgO NPs was conducted using magnesium chloride (MgCl_2_) as a reducing agent along with the plant extract of *Calotropis gigantea*. Using the floral extract of *C. gigantea*, green synthesis of MgO (G-MgO) NPs was successfully achieved and characterized, uncovering their nanoscale toxicity. Comparative cellular toxicity analysis showed higher biocompatibility of G-MgO NP compared to MgO NP with reference to the morphological changes, notochord development, and heartbeat rate in embryonic zebrafish LC50 (where 50% zebrafish were died due to lethal concentration) of G-MgO NP was 520 μg/mL compared to 410 μg/mL of MgO NP. Molecular toxicity investigation revealed that the toxic effects of MgO NP was mainly due to the influential dysregulation in oxidative stress leading to apoptosis because of the accumulation and internalization of NPs and their interaction with cellular proteins like Sod1 and p53, thereby affecting structural integrity and functionality. The study delineated the nanotoxicity of MgO NP and suggests the adoption and use of new green methodology for future production (Table 7).

## TOXICITY ISSUES AND SAFETY STUDIES OF GREEN SYNTHESIZED NANOPARTICLES

Toxicity issues for green synthetic NPs should also be identified. In prior investigations, researchers have employed various amounts of green synthesized NPs in animal models. Based on the studies described above, the optimal dose of green synthesized metal NPs was found to be 16 to 90 mg/kg [65,71,75,81] in broilers, and the optimal dose was recommended to be 10 to 16 mg/kg [76,94] in aquatic organisms based on the type of metal NPs.

However, some studies have shown that excessive amounts of green NPs can be toxic to animals, resulting in negative consequences for animal growth. Hidayat et al [71] found that broilers fed 180 mg Zn/kg as a feed additive experienced weight loss and decreased palatability. Similarly, a group at a higher dose of 0.4 mg/kg in broilers revealed that it can cause DNA damage in major organs of the body [83]. The toxicity of green synthesized NPs is still under debate. Therefore, dose-specific toxicity evaluation and safety studies in different animal species are essential to utilize safe GNT.

## CONCLUSION AND FUTURE OUT LOOK

Advances in NT have the potential to increase animal productivity and bio-efficiency, as well as improve nutritional management. However, conventional chemical synthesis methods are expensive and the release of chemicals during the synthesis process puts a strain on the environment. If these chemicals persist in animals, they have the potential to ultimately affect humans.

Green nanotechnology has recently gained attraction to address these issues. Green synthesis utilizes eco-friendly materials such as plants to replace chemicals, which can reduce toxic emissions and contribute to cost savings. It is also expected to be effective in increasing animal productivity in the livestock industry by minimizing chemical residues, which has a positive impact on animal growth and immune systems.

However, research on GNT is currently in its infancy, and quantitative and detailed animal-specific experiments are needed to accurately determine the effects on animals. In particular, systematic studies of long-term safety and environmental sustainability in different animal species and conditions are needed.

Green nanotechnology has the potential to revolutionize not only agriculture and animal husbandry, but also medicine, food, and other fields. Therefore, the research and development of GNT should be accelerated, and it will play an important role in laying the foundation for a sustainable future. Green nanotechnology will achieve environmental protection and resource efficiency at the same time and greatly improve our quality of life.

## Figures and Tables

**Table 1:** Comparison of traditionally synthesized metal nanoparticles and green synthesized nanoparticles

Parameters	Traditional metal nanoparticles	Green metal nanoparticles	Reference
Toxicity	Discharge of hazardous and toxic chemicals	Minimizes toxicity by reducing the use of harmful chemicals	[42]
Safety	Possible hazard arising from chemical reactivity or harmful properties	Improved safety attained through the utilization of eco-friendly and biocompatible substances	[43]
Energy consumption	Requires high energy input, temperature, and pressure	Relatively low energy consumption with the availability of renewable energy	[44]
Solvent usage	Frequent use of toxic chemicals and solvents	Utilizes eco-friendly solvents like ethanol and distilled water	[45,46]
Environmental impact	Emission of pollutants and challenges with waste disposal	Prevent pollution during the initial stages of chemical process and reduce negative effect on the environment	[47]
Cost	Require expensive chemicals and energy	Renewable resources and simple processing contribute to cost efficiency	[48–50]
Material efficiency	Production of hazardous waste	Utilize renewable natural resources and minimize waste generation during reactions	[51,52]

**Table 2:** Effects of green synthesized AuNPs on monogastric animal health

Type	Animal	Diet	Result	References
Fe_3_O_4_/Au magnetic nanoparticles (AuNPs) mediated by *Calendula* flower extract	Total 90 pregnant rat (210±5 g) for 25 days trial	Total 75 rats in six diet groups such as control: distilled water, negative control: untreated water, positive control: Glibenclamide at 20 mg/kg, and three groups: Au nanoparticles at 20, 40, and 80 μg/kg	Fe_3_O_4_/AuNPs: ↓GGT, ALT, AST, ALP, glucose ↑liver weight, ↑liver volume, ↔ Glibenclamide in the liver	[58]
AuNPs (*Ziziphus zizyphus* leaf extract)	8-weeks-old male Albino mice / 28 days trial	Total 30 male albino mice divided three diet groups: acute (1 g/mg i.p.), chronic (1 mg/kg i.p.), and control with no i.p. of AuNPs.	AuNPs: ↔body weight, ↑gold content (liver, spleen, kidney), ↔organ indices (liver, kidney, heart, spleen), ↔gold level in the blood and heart,	[59]
*Bacopa monniera* stabilized gold nanoparticles (*Bm*GNPs)	90-days-old male adult albino mice	Total 40 male adult albino mice with five diet groups: control, aluminum acetate (5 mg/kg b.w), *B. monniera* extract (5 mg/kg b.w), *Bm*GNPs (5 mg/kg b.w), aluminum acetate plus *Bm*GNPs	*Bm*GNPs: ↓TBARS ↑SOD, CAT, and GPx scavenging property of sGNPs.	[60]

AuNPs, gold nanoparticles; ↑, increase/upregulation; ↓, decrease/downregulation; ↔, no change; FBS, fetal bovine serum; GGT, gamma-glutamyl transferase; ALT, alanine transaminase; AST, aspartate aminotransferase; ALP, alkaline phosphatase; TBARS, thiobarbituric acid-reactive substance; SOD, superoxide dismutase; CAT, catalase; GPx, glutathione peroxidase; i.p., intraperitoneal injection.

**Table 3:** Effects of green synthesized AgNPs on monogastric animal health

Type	Animal	Diet	Results	References
Green AgNPs of *Corallina elongata* extract	One-week Ross broiler/35 days trial	Total 240 broiler with four diet groups: control, biogenic AgNPs coated with acetic acid (5 mL/L), biogenic AgNPs (5 mL/L), and finally, 5 mL/L of acetic acid	biogenic AgNPs: ↑body weight, ↔blood parameters (ALT, AST) ↓cholesterol, abdominal fat ↔carcass, ↑immune response	[64]
Oregano bioactive lipid compound (OBLC) and silver nanoparticle (AgNPs)	One-day-old Ross 308 chicks /35 days trial	Total 320 broiler chicks with four diet groups: control, control with 150 mg/kg OBLC, control with 4 mg/kg AgNPs, and control with OBLC + AgNPs	OBLC+ AgNPs: ↑body weight ↓feed conversion ratio, ↑liver function, ↓abdominal fat, ↓death rate	[65]
Green synthesized AgNPs	2-weeks-old Nile tilapia/28 days trial	Total 150 Nile tilapia divided in five diet groups: 0.00, 3.31, 6.63, 13.25, and 26.50 mg/L	Green synthesized AgNPs over 3.31 mg/L: ↓antioxidant genes (SOD, CAT) ↓enzymes, ↑MDA, ↓RBC, WBC, Hb, HCT, ↓total protein, globulin	[68]

AgNPs, silver nanoparticles; ↑, increase/upregulation; ↓, decrease/downregulation; ↔, no change; ALT, alanine transaminase; AST, aspartate aminotransferase; SOD, superoxide dismutase; CAT, catalase; GSH, glutathione; RBC, red blood cell; WBC, white blood cell; QNP, quercetin nanoparticles; AVH, abalone viscera hydrolysate; MDA, malondialdehyde; Hb, hemoglobin; HCT, hematocrit.

**Table 4:** Effects of green synthesized zinc nanoparticles on monogastric animal health

Type	Animal	Diet	Result	References
Nano Zn-Phytogenic (NZP)	One day old broiler chickens/33 days trial	Total 360 broiler chickens with six diet groups: basal diet; basal + Zn Sulfate (90 mg/kg) + 5.32 mg/kg guava leaf meal (added as a source of phytogenic compounds); basal + NZP (45 mg/kg); basal + NZP (90 mg/kg); basal + NZP (135 mg/kg) and basal + NZP (180 mg/kg).	NZP at 90 mg/kg body: weight gain ↑ FCR ↔ SOD activity ↑ Pathogenic bacteria↓ (*E. coli*, *Salmonella* sp.)	[71]
Zinc oxide nanoparticles (ZnONPs) with Nigella sativa	A day old broiler chicks	Total 150 broiler, five diet groups: control negative: uninfected and untreated, control positive: infected and untreated; 3rd, 4th and 5th groups were infected orally with 5×10^4^ sporulated oocysts of *Eimeria tenella* and treated with 60 mg/kg ZnONPs, 1% *Nigella sativa* seeds and amprolium 125 ppm, respectively	ZnONPs with *Nigella sativa*: ↓pro-inflammatory cytokine (IL-2, TNF-α) ↑anticoccidial, ↑antioxidant, ↑anti-inflammatory ↑growth performance	[72]
Biosynthesized zinc oxide nanoparticles	One day old broiler chickens for 35 days trial	Total 180 broiler chicks (Cobb500) with five diet groups: basal diet with 100 mg/kg ZnO (control) and 10, 40, 70, and 100 mg/kg ZnO NPs	ZnO NPs (at 70 and 100 mg/kg): absorption↑, bone mineralization↑, bone weight, length, and thickness ↑ antioxidative status in serum and liver tissues↑,	[74]
Green nano zinc (GNZ)	Broiler chicken for 42 days trial	Total 432 broiler chicken nine diet group; three levels (40, 60, and 80 ppm) and three sources (inorganic, green nano, and market nano) of zinc.	GNZ at 80 ppm: bone dimensions ↑ weight, total ash ↑ phosphorus ↑ (SOD, glutathione peroxidase, catalase, zinc, calcium level )↑ fat, cholesterol ↓	[75]
ZnONPs green synthesis with plant, *Spinacia oleracea*	Freshwater fish, *Labeo rohita* for 45 days trial	Green synthesized ZnONPs (5, 7.5, and 10 mg/kg)	Green synthesized ZnONPs: ↑growth performance ↑biochemical, hematological, digestive enzyme activities	[76]
*M. pulegium* extract green zinc nanoparticles (GZnO-NPs)	Mucus of goldfish for 2 weeks trial	Total 225 male *C. auratus* with five diet groups: Control (without ZnO-NPs), T1 (0.9 mg/L ZnO-NPs), T2 (1.9 mg/L ZnO-NPs), T3 (0.9 mg/L GZnO-NPs), T4 (1.9 mg/L GZnO-NPs).	GZnO-NPs at 0.9 and 1.9 mg/L: ↑total protein, albumin, hemoglobin, ↓glucose, cortisol, ALT, AST, MDA, urea, creatinine ↓oxidative damage	[77]

ZnNPs, zinc nanoparticles; ↑, increase/upregulation; ↓, decrease/downregulation; ↔, no change; BWG, body weight gain; ALT, alanine transaminase; AST, aspartate aminotransferase; LDH, lactate dehydrogenase; FCR, feed conversion ratio; SOD, superoxide dismutase; CAT, catalase; GPx, glutathione peroxidase; MDA, malondialdehyde; Ig, immunoglobulin; HGB, hemoglobin; IL, interleukin; TNF-α, tumor necrosis factor-alpha.

**Table 5:** Effects of green synthesized selenium nanoparticles on monogastric animal health

Type	Animal	Diet	Results	References
Green nano selenium (GNS)	One day old ross 308 male broiler chickens /42 days trial	Total 360 male broiler chickens four diet groups (1) based diet group, (2–4) basal diet with 0.075, 0.15 and 0.3 mg/kg of GNS, respectively.	In 0.3 mg GNS/kg: ↔growth performance ↑Se concentration ↑lactic acid bacteria counts, ↑lactic acid bacteria/coliform ratios in ileum, ↑breast and thigh muscles, ↑IgG	[80]
Green nano selenium (GNS)	Broiler chicken/42 days trial	Total 432 broiler chicken nine diet groups: IS-0.15, GNS-0.15, MNS-0.15, IS-0.20, GNS-0.20, MNS-0.20, IS-0.25, GNS-0.25, and MNS-0.25	In GNS 0.25 ppm: ↑growth performance, ↑immune response, ↑lymphoid organ development, ↑Se concentration (liver, breast muscle) ↑nitrogen digestibility, ↑serum antioxidant activity,	[81]
Nano-Se using scent leaf (Ocimum gratissimum) extract	One day old broiler chicken/35 days trial	Total 200 Arbor acre broiler with chickens five diet groups: control which had 0 levels of nano Se while treatments 2, 3, 4 and 5 had 0.10, 0.15, 0.20 and 0.25 mg/kg levels of nano Se	Nano-Se at all levels: Growth performance↑, Immunity↑, Nutrient digestibility↑	[82]
Se-NPs using *Capsicum annum* extract	One day old Japanese quails/35 days trial	A total of 480 Japanese quails with three diet groups: control and others received 0.2 mg/kg and 0.4 mg/kg of se-NPs via oral gavage, respectively	SeNPs at 0.2 mg/kg: ↓ FCR ↔feed intake, ↑ weight gain, high SeNPs at 0.4 mg/kg: ↑ WBC and ↓ RBC, Hb	[83]

SeNPs, selenium nanoparticles; ↑, increase/upregulation; ↓, decrease/downregulation; ↔, no change; Ig, immunoglobulin; IS, inorganic selenium; MNS, market nano selenium; FCR, feed conversion ratio; WBC, white blood cell; RBC, red blood cell; Hb, hemoglobin.

**Table 6:** Effects of green synthesized copper nanoparticles on monogastric animal health

Type	Animal	Diet	Result	References
Nano-CuO using basil extract	1-day-old broiler chicks (Ross 308)/35 days trial	Total 96 broiler with two diet groups: a control diet or a control diet supplemented with green synthesized of Nano-CuO (8 mg/kg).	Green Nano-CuO: ↑body weight, ↑daily body weight gain ↓feed conversation ratio ↔feed intake ↑dressing percentages, ↓abdominal fat percentages, ↔AST, ALT, urea, creatinine	[86]
CuNPs using *Capparis spinosa* extract	BALB/c mice weighing 25-30 g/2 weeks trial	Total 32 male BALB/c mice with four diet groups: normal, CuNPs at 1,000, 2,000, and 5,000 μg/kg	CuNPs at all levels: ↔hematological parameters, ↔liver functions	[87]
Green nano copper (GNC)	Day old chicks/42 days trial	Total 480 chicks, 12 diet groups: the experimental design was 3x4 factorial with three levels of Cu (8, 12, 16 ppm) and four sources (IC, OC, GNC, and MNC)	At 16 ppm GNC: ↑body weight gain, ↑feed intake, ↑feed efficiency, ↑immune response, ↔Cu level, carcass ↑PHAP	[88]
CuNPs using Neem leaf extract	One day old Vanaraja chicken /56 days trial	Total 324 chicks with six diet groups: control, basal diet supplemented with 12 mg Cu/kg, and four groups fed with green synthesized nano copper at 3, 6, 9 and 12 mg Cu per kg diet, respectively.	50% copper replaced with nanocopper at 6 mg/kg: ↑ immunity, ↑ serum parameters ↑ antibody ↑ antioxidant status	[89]

CuNPs, copper nanoparticles; ↑, increase/upregulation; ↓, decrease/downregulation; ↔, no change; ALT, alanine transaminase; AST, aspartate aminotransferase; IC, inorganic copper; OC, organic copper; GNC, green nano copper; MNC, market nano copper; PHAP, phyto-hemagglutinin.

**Table 7:** Effects of green synthesized manganese nanoparticles on monogastric animal health

Type	Animal	Diet	Results	References
MnO_2_NPS (green tomato extract)	One-day-old Arbor broiler chicks/ 35 days trial	Total 150 broiler chicks with five diet groups: basal diet (60 mg/kg MnO_2_), basal diet with an additional 66 mg/kg MnO_2_, basal diet with 72 mg/kg MnO_2_, basal diet with 66 mg/kg MnO_2_ NPs, basal diet with 72 mg/kg MnO_2_ NPs	Green synthesized MnO_2_NPS: ↑Body weight, ↑body weight gain ↓feed conversation ratio ↑SOD, ↑GSH, GST,	[93]
Nano Mn_3_O_4_ using *Ananas comosus* (L.) peel extract	Prawns /90 days trial	Total 840 PL (post larvae) and seven diet groups: 0 (control), 3.0, 6.0, 9.0, 12, 15 and 18 mg/kg dry feed weight.	Nano Mn_3_O_4_ at 16 mg/kg: ↑growth performance (final growth, weight gain) ↑digestive enzyme activities ↑muscle biochemical compositions, ↑total protein level. ↔antioxidants enzymatic activity (SOD, CAT) ↔hepatopancreas	[94]

MnNPs, manganese nanoparticles; ↑, increase/upregulation; ↓, decrease/downregulation; ↔, no change; SOD, superoxide dismutase; CAT, catalase; GSH, glutathione; GST, glutathione S-transferase.
